# Public health consequences of global climate change. A narrative review

**DOI:** 10.3389/fcimb.2026.1794031

**Published:** 2026-06-24

**Authors:** Anna Apetrei-Pandrea, Stefan Dascalu

**Affiliations:** 1 School of Public Health, University of Pittsburgh, Pittsburgh PA, United States; 2 Kenneth P. Dietrich School of Arts and Sciences, University of Pittsburgh, Pittsburgh, PA, United States; 3 Department of Biology, The University of Oxford, Oxford, United Kingdom

**Keywords:** carbon emissions, climate change, climate denialists, emerging infections, public health, re-emergent infections, vector-borne diseases

## Abstract

Human activities related to the industrialization generated an overall rise of global temperatures with approximately 1 °C since 1880. These increases in temperatures have been accelerating since 1981, with the rate of temperature increase roughly doubling since the 1970s, the average warming rate being of approximately 0.18 °C to 0.20 °C per decade between 1970 to 2015. Even more alarmingly, recent studies indicate that this rate has accelerated to over 0.35 °C per decade since 2015, roughly the double of the previous period. The primarily contributor to the observed extensive climate change is the accumulation of greenhouse gases in the atmosphere, the effect of which is a significant rise in Earth’s surface temperature. Here, we examine the interplay between global warming and climate change, emphasizing their interconnected yet distinct roles. The consequences include extreme weather events, permafrost melting, and biodiversity loss. Most importantly, climate change represents a direct threat for human health. Higher temperatures can produce metabolic imbalances and oxidative stress, that may be responsible for various levels of immunosuppression, increased susceptibility to infections, and ultimately death. Climate change is proven to be associated with increased frequency and emergence of vector-borne diseases, mainly due to significant expansion of the endemic areas for the vectors. It is also related to exacerbation of respiratory, cardiovascular, and infectious diseases and an increase in the prevalence of psychiatric disorders. Given the limitations of climate change modeling, proactive policies and adaptive strategies are imperative. Climate change and global warming should be central aspects of current education, and educational programs should be implemented at every societal level. Actions to control climate change need to be continuously adapted to the observed reality and, should the current targets be deemed as insufficient to address the main problems, new, more ambitious, goals have to be negotiated and implemented. Solving the complex challenge of climate variability will necessitate a coordinated and sustained global action, independent of political views, geographical location and individual interests to safeguard both environmental and public health.

## Introduction

There is a general consensus that mankind is facing significant climate change, triggered by human activities related to the Industrial Revolution, and resulting in an overall increase of global temperatures of about 1 °C (about 2°F) since 1880. These increases in temperatures have been accelerating since 1981, with the rate of temperature increase roughly doubling since the 1970s, the average warming rate being of approximately 0.18 °C to 0.20 °C per decade between 1970 to 2015 (Centers for Diseases Control and Prevention, 2026; World Health Organization, 2026). Even more alarmingly is the fact that recent studies indicate that since 2015, this rate has accelerated to over 0.35 °C per decade, roughly the double of the previous period, making countering of the global warming a worldwide priority (Lindsey et al., 2025).

**Figure 1:**
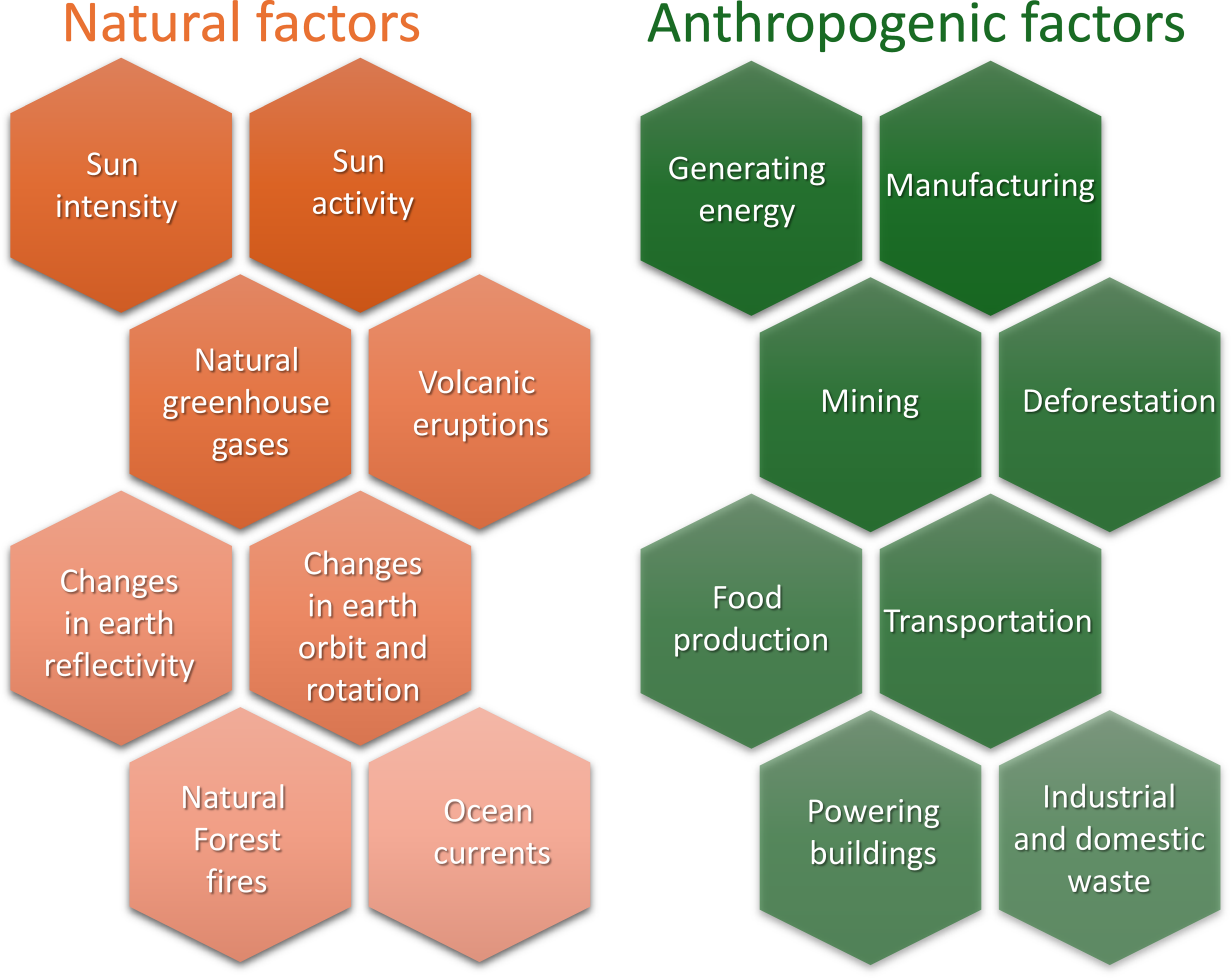
The drivers of climate change can be grouped into two categories: natural factors, which are responsible for the rhythmic cycles of climate change that occurred throughout Earth’s history. These factors cannot be controlled, but in the meantime, they did not decisively contribute to the accelerated climate change that was seen during the last two centuries; meanwhile, anthropogenic factors, i.e., man-driven determinants of climate change were instrumental to the recent climate change, with severe consequences for public health and their impact has the potential to be irreversible. Moreover, anthopogenic factors can be controlled through global actions and regulations. Regulations of the anthropogenic contribution to climate change will also have a major impact on global health. As illustrated, the action of natural and anthropogenic factors is partially intricated.

While the public in vernacular language uses “global warming” and “climate change” as interchangeable terms (Lynas et al., 2021), they are not 100% superimposable, referring to slightly different concepts (National Aeronautics and Space Administration, 2024). Global warming is the gradual, long-term increase in the Earth’s surface temperature triggered by emissions and accumulations of greenhouse gases from human activities, i.e., carbon dioxide (CO_2_), methane (CH_4_), and nitrous oxide (N_2_O). The “*greenhouse effect*” exerted by these accumulated gases traps heat in the atmosphere, thus leading to planetary warming (Mann and Encyclopedia Britannica, 2024). Meanwhile, the term climate change covers all the long-term changes in the Earth’s climate systems; these can be both human-driven (anthropogenic), but in the meantime may result from natural causes and are represented by (**Figure 1**): shifts in patterns of temperature, altered precipitation patterns (e.g., storms, droughts), wing changes in wind and modifications of other climate factors (e.g., ocean acidification, sea level rises), as well as global warming. These alterations may occur over timescales ranging from decades to millions of years (Jackson and Encyclopedia Britanica, 2024).

Contemporary climate change may dramatically impact the human ecosystem as well. The sea level rises, due to both the polar ice caps melting and to water volume increases (dilatation due to higher global temperatures), and this process will result in submergence of major coastal cities. Glaciers are rapidly retreating worldwide at a melting rate substantially higher than previous projections (Glick, 2018). Recent syntheses reveal that cryospheric responses, such as shifting avalanche patterns, exemplify how interconnected atmospheric, hydrological, and ecological systems are reacting to accelerated global warming, underscoring the broad reach of climate impacts (Eckert et al., 2024). The permafrost, which currently covers a surface equivalent to that of the United States, China, and Canada combined, is melting faster than over the last 3 million years and shrunk by 10% compared to the beginning of the 20^th^ century. It is estimated that every temperature increase with 1 °C will melt 1.5 million square miles of permafrost (Chadburn et al., 2017). This poses a global threat because the permafrost is the greatest store of the greenhouse gases: (i) it can capture twice more CO2 than it exists in the atmosphere; (ii) it also captures significant amounts of methane, which traps 80% more heat than the CO2. It is estimated that, by the year 2100, permafrost melting will release an amount of greenhouse gases equivalent to 1/5 of the total emissions recorded since the beginning of the Industrial Revolution (Chadburn et al., 2017).

All these atmospheric changes further alter the climate: rainfall regimens are gradually changing and ocean temperatures are increasing. For example, in the summer of 2023, the ocean temperature around Florida reached record high values, being compared to hot tub temperatures. Climate change can also trigger wildfires that severely alter forest ecosystems, thereby reinforcing the feedback loop that drives extreme climate conditions (Tanaka and Van Houtan, 2022). These changes are happening now and it is not surprising that already, over the last two decades mankind was confronted with some of the most severe hurricanes on record (Weinkle et al., 2018). The more frequent occurrence of such extreme weather events can significantly impact our energy supply, through recurrent and frequent disruptions to energy infrastructure: power outages, damage to power lines, and reduced energy production at power plants (Gernaat et al., 2021). While models suggest that these changes will not be limited to a specific geographical area, but will rather impact the entire planet, the effects of this changing climate are pleiotropic and unequally distributed, with the most catastrophic effects of climate change disproportionately affecting those in developing nations, which have historically polluted less than the wealthy nations (Resnik, 2022).

## Methodology

This is a narrative review aiming to synthesize and critically discuss current evidence on the medical and public health implications of climate change. We conducted a targeted literature search between January 2024 and September 2025 across the PubMed, Scopus, and Web of Science databases, using combinations of the following keywords: “*climate change*,” “*global warming*,” “*public health*,” “*vector-borne diseases*,” “*emerging infections*,” “*environmental health*,” and “*climate policies*”.

Only peer-reviewed articles written in English were selected and compiled into the EndNote software, where we formed an exhaustive library. In the library we did not include any article from predatory journals. From this library, we prioritized original studies and reviews published between 2010 and 2024. Only foundational works published outside of the time frame selected were included where necessary for historical or conceptual context. The articles were cross-checked with review articles and book chapters.

We selected recent policy documents addressing public health implications of climate change based on the information found on institutional web sites. Also, we selected international reports available from major national and international agencies. When web sites were used, we selected only governmental sites (i.e., Centers for Diseases Control and Prevention-CDC or Environmental Protection Agency-EPA, National Oceanic and Atmospheric Administration in the US) and sites of international organizations (i.e., World Health Organization-WHO). These sites were also used as sources for national and international policies. During the preparation stage of this material, we had to adapt the manuscript, as government sites in the United States abruptly changed their policies and therefore their information pages. Thus, for instance, information regarding climate change completely disappeared from the EPA site in 2025, and the manuscript had to be updated accordingly.

Since this is a narrative review, we did not perform quantitative meta-analyses or formal quality appraisal. Instead, the evidence was synthesized and integrated by considering findings across relevant studies to highlight consistent trends, major uncertainties, and implications for public health and clinical practice. Also, a key criterion in synthesizing the data was the easy comprehensibility of the presented notions, with the overarching goal of increasing the addressability of the paper and make data accessible for and easy to absorb by a broad readership, with elementary background in environmental and public health sciences.

## Determinants of climate change

The key driver of global warming is the accumulation in the atmosphere of gases with a greenhouse effect: CO_2_ (which traps sun heat and prevents it from being release in space), methane, nitrous oxide, water vapor, and fluorinated gases. While greenhouse gases are normal components of the atmosphere, their concentration have increased substantially in recent years with anthropogenic activities (ex., the atmospheric levels of CO_2_ are currently 48% higher than its preindustrial levels (Adepoju et al., 2023)).

In addition to human activities, natural cycles of geological changes may lead to natural modifications in the concentration of the greenhouse gases, climate change and global warming: volcanic eruptions; the Sun’s intensity and variations in solar activity; variations in Earth’s orbit and rotation; or fluctuations in Earth’s reflectivity (**Figure 1**) (Knight and Harrison, 2013; Adepoju et al., 2023). Note that, while these natural factors contributed to the cycles of warming and cooling throughout the Earth’s history, they do not decisively contribute to the rapid heating of the Earth initiated 200 years ago by the industrial revolution (Knight and Harrison, 2013; Rantanen et al., 2022; Henley et al., 2024).

The scientific consensus is that human behavior is the root cause of the current cycle of global warming (**Figure 1**) (Tian et al., 2016; Gong et al., 2024). The use of fossil fuels is by far the biggest contributor to global warming, through the emission of greenhouse gases in large amounts (Adepoju et al., 2023; Supran et al., 2023; Filonchyk et al., 2024b). A 2024 global assessment estimated total greenhouse gas emissions at approximately 53.8 GtCO_2_-eq, of which the power sector (27.5%), transportation (15.1%), and industrial combustion (12.2%) together account for over half of total anthropogenic output, pinpointing priority areas for mitigation (Filonchyk et al., 2024a). Additionally, agriculture (particularly animal production and farming on a large scale) is a major contributor to the atmospheric methane (Adepoju et al., 2023; Worku, 2024). Fertilizers release nitrous oxide in the air. Meanwhile, modern agricultural hardware run on fossil fuels, which generate additional emissions (Lynch et al., 2021). Atmospheric CO_2_ sequestration is limited by deforestation, while wood burning or decay increases nitrous oxide release into the atmosphere (Bala et al., 2007). Deforestation is also responsible for the emergence of new pathogens into the human population (Olivero et al., 2017; Tazerji et al., 2022). Mining releases sequestrated gases and also increases interactions between humans and bats, which carry a plethora of viruses, to which they appear to be tolerant (Pei et al., 2024; Roffler et al., 2024; Shousha et al., 2024). Other sources of greenhouse gases are the domestic wastes of the appliances that use freon (refrigerators, air conditioners, heat pumps) and the industrial machines, which are powered by fossil fuels (Adepoju et al., 2023).

## Impact on human health

**Figure 2:**
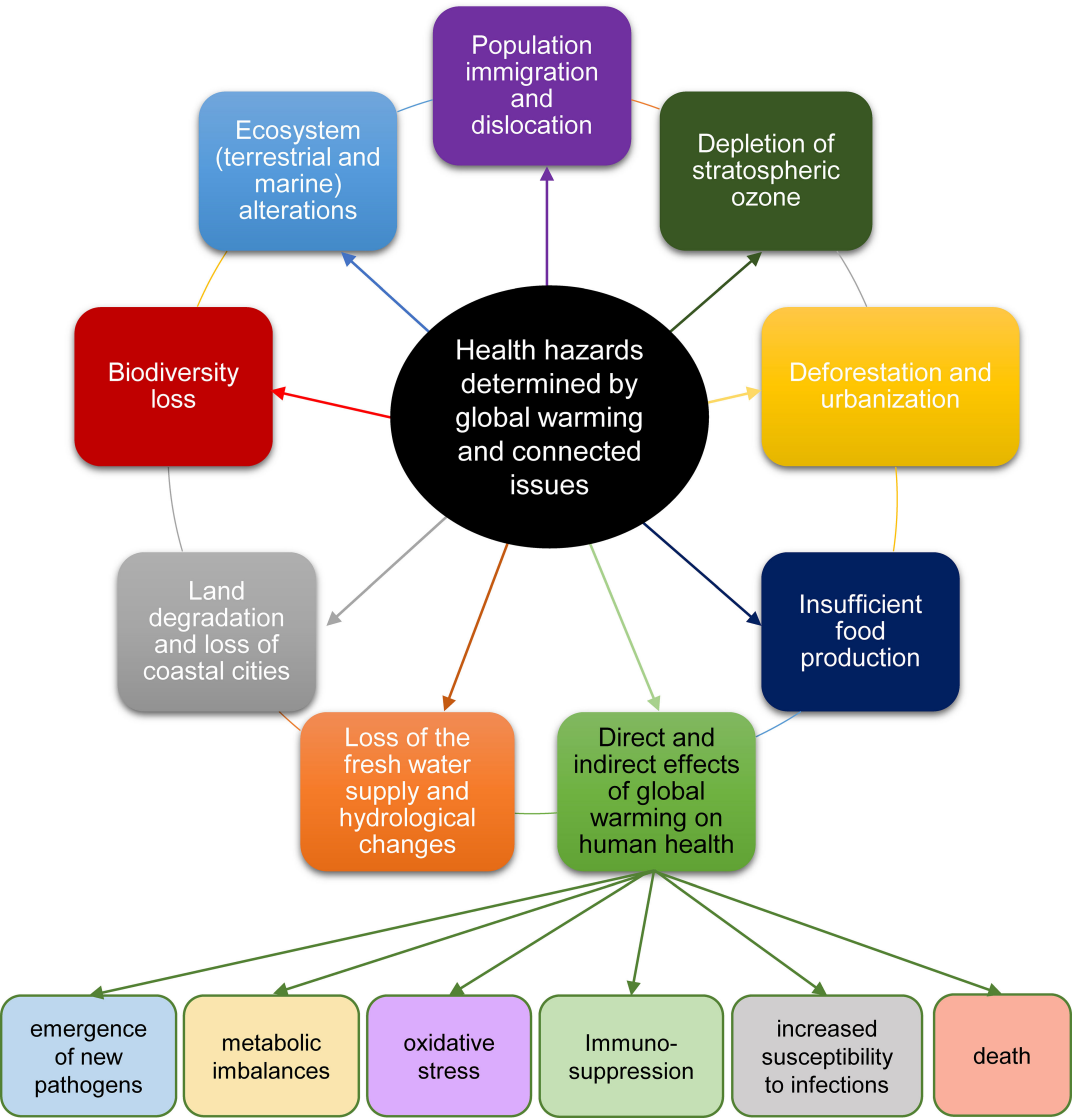
Direct and indirect health hazards due to climate change and its consequences. Climate change, through increases in temperatures can exert a direct impact on human health (and increase oxidative stress, alter metabolic balances, induce immunosuppression and this increase susceptibility to infections and the overall risk of death). Additionally, through modifications in the human ecosystems (i.e., loss of biodiversity, land degradation and increase in sea levels, depletion od stratospheric ozon, deforestation or loss of food and water supplies) climate change exert an indirect effect on human health, increasing the risk factors for a plethora of diseases.

The World Health Organization (WHO) identifies climate change as one of the greatest threats to global health (World Health Organization, 2026). Climate change may impact health directly or indirectly (**Figure 2**), with both being triggered by the increased temperatures due to global warming and the consequential higher frequency and intensity of heatwaves. Higher temperatures can produce metabolic imbalances and oxidative stress, that may be responsible for various levels of immunosuppression, increased susceptibility to infections, and ultimately death, as illustrated by the higher mortality for dairy cattle occurring worldwide in the summer days with recorded heatwaves (Chadburn et al., 2017). The heat-associated pathologic conditions equally affect humans (Agache et al., 2022): thus, an extreme heat wave that occurred during the summer of 2003 led to the demise of 35,000 Europeans, while associating excessive domestic animal loss in at least two regions of France (Adepoju et al., 2023). Recent studies estimated that global warming has doubled the probability of recurrence of similar heat waves (Agache et al., 2022). Thus, the extreme heat wave that confronted Europe in 2022, associated an estimated 61,672 heat-related excess deaths (WHO). In India, heatwaves with temperatures above the 97th percentile for two consecutive days associate a 14.7% increase in daily mortality (de Bont et al., 2024). It is also estimated that the highest mortality from heatwaves mostly occurs in Asia and Europe, with 178,486 estimated global excess deaths being recorded during the hottest days in 2023 (de Bont et al., 2024).

Another direct mechanism through which climate change driven by global warming can destroy human and animal life is represented by the extreme weather events (Ebi et al., 2021; Khraishah et al., 2022). As climate change occurs, the frequency and intensity of hurricanes, cyclones, typhoons and floods increase (Bell et al., 2018; Ebi et al., 2021); these catastrophic events severely impacted over 95 million people and were responsible for 11,755 deaths in 2019 (Ebi et al., 2021).

Global warming may also result in a degradation of air quality, which is an additional health hazard, contributing to an increased incidence of respiratory diseases (Gherasim et al., 2024). A temperature rise favors the reaction between industrial or anthropogenic pollutants and sunlight, and the formation of the ozone smog. Note, however, that ozone formation may have some additional causes, being driven by the increased temperature, water vapor levels, and air circulation patterns. Once inhaled, ozone causes inflammation of the lungs, generating respiratory complications and aggravating pre-existing respiratory diseases (Kline and Prunicki, 2023; Lichtblau et al., 2024).

Increased exposure to ultraviolet radiations triggers dermatologic diseases, with a dramatic increase in the incidence of skin cancer being observed during the second half of the 20^th^ century (Belzer and Parker, 2023).

There are also several indirect ways through which climate change driven by global warming influence human and animal health: (i) eutrophication (i.e., excessive richness of nutrients, due to runoff from the land and lack of oxygen that may trigger excessive plant growth and animal death); (ii) the growth of harmful algae, which may compromise the quality of potable water (Li et al., 2023); (iii) expansion of the duration of plant polenization due to longer warm season, with increased incidence of allergies and asthma (Li et al., 2023); (iv) the overgrowth of the mycotoxin-producing fungi on cereals, favored by higher temperatures may lead to an increased incidence of allergic diseases. Mycotoxins can be ingested by animals extensively fed with cereals, or by humans, through wheat consumption. Pollutant exposure is also triggering a large spectrum of neurological diseases: white matter disease, amyloidosis, cognitive deficiencies, stroke, increased risk for transient ischemic attacks, migraine, increased risk for dementia, vascular dementia, and brain atrophy (Louis et al., 2023).

Deforestation and mining can impact human health indirectly, by allowing vectors that carry pathogens to emerge into the human population, following the disruption of their natural habitats by human activities. Human exposure is increased not only through the release of the pathogens from their ecosystems, but also by their increased ability to persist and to be transmitted to humans in the context of the new climatic paradigms (Burkett-Cadena and Vittor, 2018; Tazerji et al., 2022; Shousha et al., 2024). Human exposure to pathogens carrying deadly viruses may also increase through bush meat consumption (Peeters et al., 2002; Ahuka-Mundeke et al., 2012; Liegeois et al., 2012; Worobey et al., 2022; Crits-Christoph et al., 2024).

Finally, it is also estimated that an increased climate stress will generate a significant increase in social and domestic violence, the targets of which will be women originating from disadvantaged populations. This additional domestic stress will result in a massive increase of mental and reproductive health problems (Segal and Giudice, 2022; Sri et al., 2023; Woodland et al., 2023).

One of the few benefits of global warming is that, by reducing the overall number of cold days and by increasing the overall winter temperatures, it may limit cold-related mortality (Li et al., 2023). However, any limited reduction in cold-related mortality is greatly outweighed by the broader adverse health effects of climate change.

## Emergence and re-emergence of infectious diseases

The combination between increased temperatures and alterations of the precipitation patterns due to climate change creates favorable conditions for the emergence and re-emergence of multiple infectious diseases (**Table 1**) (Weaver, 2013; de Souza and Weaver, 2024). Thus, in 2023, seven cases of malaria (six in Florida and one in Texas) were reported to occur in two unrelated outbreaks in persons without a history of international travel, raising concerns of malaria re-emergence in the United States (Centers for Disease Control and Prevention, 2023). This is the first time that the disease was observed in Florida in 20 years (Centers for Disease Control and Prevention, 2023). Malaria used to be endemic in the Southern United States, but it was eliminated in 1951 following a massive effort involving generalized implementation of insecticides, drainage ditches, and window screens (Dye-Braumuller and Kanyangarara, 2021). The occurrence of the current cases created concern of whether or not this new outbreak resulted as a shift in the exposure to the mosquito vectors of malaria due to climate change.

**Table 1:** Major vector-borne diseases impacted by climate change.

Disease	Pathogen (Type)	Primary vector	Main endemic/emerging regions	Climate sensitivity/mechanism	Refs.
Malaria	*Plasmodium* spp. (parasite)	*Anopheles* mosquitoes	Sub-Saharan Africa, South Asia, South America; re-emerging in Southern Europe and Southern U.S.	Temperature and rainfall determine mosquito survival and parasite development rate	(Weaver, 2013; Dye-Braumuller and Kanyangarara, 2021; de Souza and Weaver, 2024)
Dengue	*Dengue virus* (Flavivirus)	*Aedes aegypti*, *A. albopictus*	Tropical/subtropical regions; expanding into Southern Europe and U.S. Gulf Coast	Higher temperatures shorten vector incubation; increased rainfall expands breeding sites	(Pando-Robles et al., 2025; Pham et al., 2025)
Chikungunya	*Chikungunya virus* (Alphavirus)	*Aedes aegypti*, *A. albopictus*	Africa, Asia, Southern Europe	Warmer temperatures accelerate vector development; altered precipitation patterns affect outbreaks	(Tjaden et al., 2017; Rocklov et al., 2019)
Zika	*Zika virus* (Flavivirus)	*Aedes aegypti*	Americas, Southeast Asia, Africa	Temperature and humidity enhance transmission efficiency	(Paz and Semenza, 2016; Yang and Sarfaty, 2016)
West Nile Virus	*West Nile virus* (Flavivirus)	*Culex* mosquitoes	North America, Europe, top East	Increased temperatures enhance viral replication in vectors	(Nash et al., 2001; Samy et al., 2016; Paz, 2019; D'Amore et al., 2022; Wang HR. et al., 2024)
Leishmaniasis	*Leishmania* spp. (protozoa)	*Phlebotomus* sandflies	South America, Mediterranean, top East	Rising temperature and urbanization increase sandfly habitats	(Trájer, 2025)
Chagas Disease	*Trypanosoma cruzi* (protozoa)	Triatomine (“kissing” bugs)	Latin America; emerging in Southern U.S.	Temperature and humidity affect insect survival and distribution	(Jones et al., 2008)
Tick-borne Encephalitis (TBE)	*TBE virus*	*Ixodes ricinus* ticks	Northern and Central Europe	Milder winters expand tick season and geographic range	(Jones et al., 2008)
Lyme Disease	*Borrelia burgdorferi* (bacterium)	*Ixodes* ticks	North America, Europe	Warmer temperatures extend tick activity and distribution	(Jones et al., 2008)

Shifts in temperature were already reported to contribute the “resuscitation” of old pathogens. Thus, in 2016, in the polar regions, permafrost melting triggered contagion with an ancient anthrax strain released from a frozen reindeer. Such re-emergence of old pathogens might pose risks to humans due to lack of immune protection to very old microbe variants (Stella et al., 2020).

While these situations are impressive, making for breaking news, they represent only a small fraction of the real issues of the climate-related emergence and re-emergence of infectious diseases, particularly vector-transmitted diseases, which constitute a very real and concerning public health consequence of climate change. The malaria outbreaks mentioned above stem from the overall warming of the planet that expands to the habitat of the malaria vectors, the *Anopheles* spp. mosquitoes, into the subtropical areas, such as the South of the United States, Southern Europe, subtropical regions from South-East Asia, or the highlands of Kenya and the Andes (Pinault and Hunter, 2011; Afrane et al., 2012; Gebhardt et al., 2022; Carlson et al., 2023). In the meantime, the new climate conditions allow the multiplication of better adapted insect vectors. These vectors not only gradually expand their habitat, but they also migrate to previously nonendemic areas, the consequence being a significant expansion of the endemic areas for vector-borne diseases. Disease-transmitting insects have expanded significantly over the recent years, being actually responsible for the emergence of new pathogens and new outbreaks or even pandemics, as was the Zika pandemic (Paz and Semenza, 2016; Yang and Sarfaty, 2016). In the meantime, numerous existent vector-borne diseases, i.e., Chagas disease, leishmaniasis, chikungunya and dengue, have recently surfaced outside of their normal endemicity areas, being reported, for example, in Southern Europe and Southern states of the US (Weaver, 2013; Hotez, 2016).

The vast majority of vector-transmitted diseases are spread by arthropods (i.e., mosquitoes, flies, and ticks). These vectors are unable to independently maintain their optimal body temperature for growth and reproduction, and rely on the ambient temperatures for this process. Higher temperatures and humidity favor the vectors, and this is the main reason for which the vast majority of cases of vector-borne diseases are recorded at tropics, being historically much easier to control such diseases in the temperate regions (Hays, 2005; de Souza and Weaver, 2024). Global increase in temperature caused by climate change triggered a vector redistribution and of the vector-borne diseases (Tjaden et al., 2017; Colón-González et al., 2021) through shifts in the balance between the warm and cold days of the year, and an overall warming above the oceans, particularly over the arctic. These vectors can transmit numerous viruses and parasites, which are at the origin of most of the major tropical diseases, such as malaria (Plasmodia), yellow fever (yellow fever virus), West Nile virus infection (West Nile virus), Chikungunya (Chikungunya virus), dengue (dengue virus), Zika (Zika virus), Japanese encephalitis (Japanese encephalitis virus), tick-borne encephalitis (tick-borne encephalitis virus), Lyme disease (*Borrelia burgdorferi*), the agent of African trypanosomiasis (*Trypanosoma brucei*), Chagas disease (*T. cruzi*), filarias (*Filaria* spp.), leishmaniasis *(Leishmania* spp.), schistostomiasis (*Schistostomas* spp.*)*, dracunculiasis (*Dracunculus medinensis*) and onchocerciasis (*Onchocerca volvulus*). Each year, these vector-borne pathogens cause diseases that are responsible for significant morbidity and mortality at a global level (Samy et al., 2016; World Health Organization, 2017; Minigan et al., 2018; Estrada-Peña and Fernández-Ruiz, 2020; Mordecai et al., 2020).

**Figure 3:**
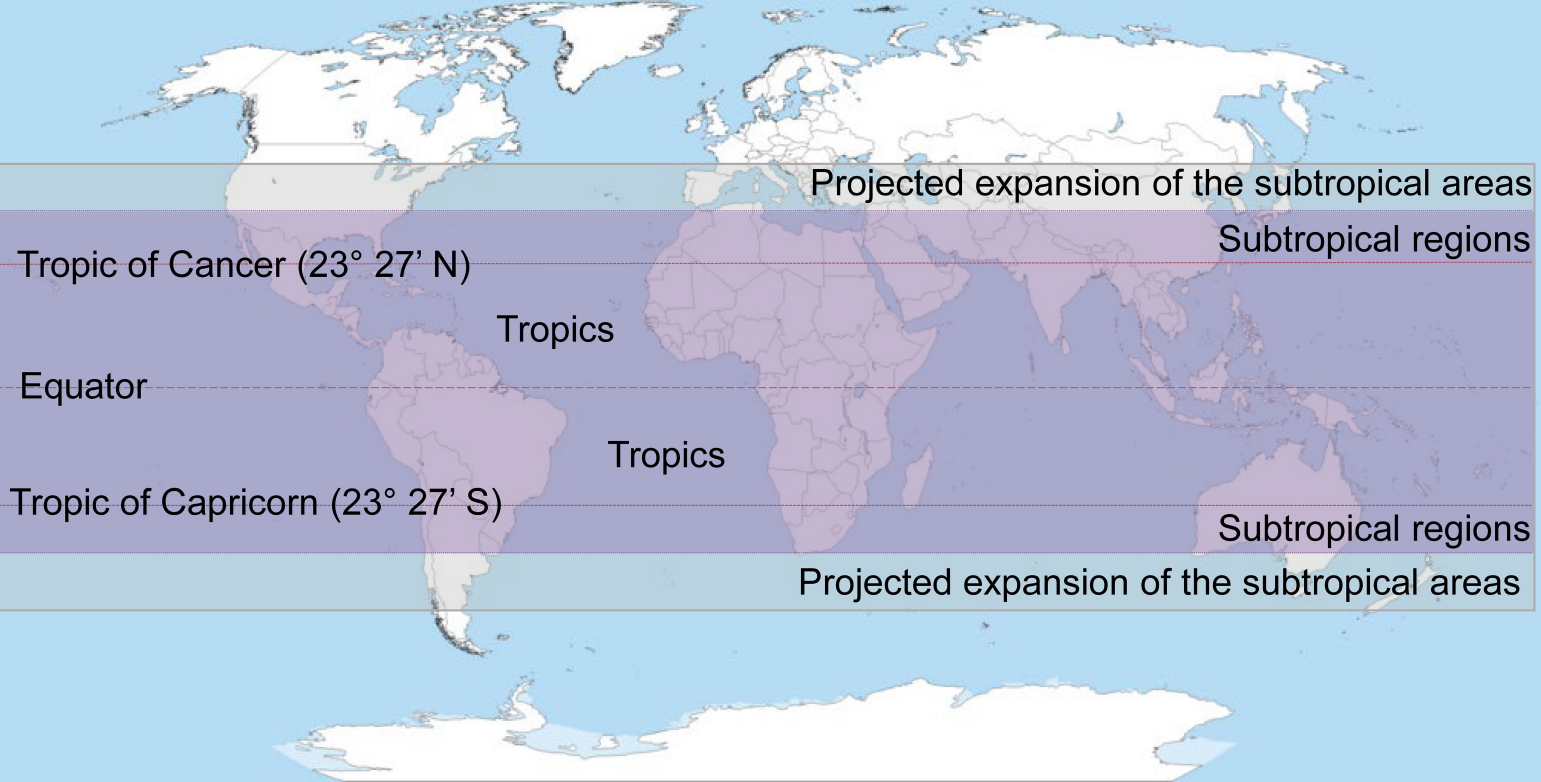
Impact of the climate change on the potential distribution of the vectors responsible for major vector-borne diseases. Multiple vector-borne infectious diseases and their vectors, such as malaria (spread by *Anopheles* mosquitoes); yellow fever, dengue, Zika, Chikungunya, Rift Valley fever (spread by *Aedes* mosquitoes); Japanese encephalitis, lymphatic filariasis, and West Nile fever (spread by *Culex* mosquitoes) are currently spread in the tropical and subtropical climate (highlighted in light violet). An increase in global temperatures has a very strong potential to expand their distribution into the temperate regions (highlighted in grey), where temperatures may reach levels suitable for vector expansion and disease transmission.

As the warming process progresses, increasing areas of subtropical and temperate regions become climate-friendly for vectors that would normally only survive in the tropics (Jones et al., 2008; Ryan et al., 2019) (**Figure 3**). Vectors that expand to new ecosystems carry pathogens associated with tropical diseases, expanding their distribution. This process is widely acknowledged as being one of the major threats generated by global warming.

The relation between vector expansion and epidemic outbreaks was best documented during the Zika epidemic. While cases were imported worldwide from the endemic regions, secondary transmissions only occurred in areas where the vectors (*Ae. Albopictus* and *Ae. Aegypti)* were endemic. The only exception to this rule are the Hawaiian Islands, where Aedes spp. mosquitoes are circulating but no cases of Zika has been yet recorded. Thus, in the US, secondary cases were recorded in Texas and Florida, where both *Ae. Albopictus* and *Ae. Aegypti* are present (Olson et al., 2020). Note that these are also the states that reported local spread of dengue and Chikungunya (Stephenson et al., 2022; Vaughan et al., 2026; Xu et al., 2026). In the past the South of the United States was confronted with some of the most severe yellow fever outbreaks in history (Tomlinson and Hodgson, 2005; Curtis, 2008).

During the Zika pandemic, cases imported from French Polynesia were documented in France (Lazarus et al., 2016). Continental France also reported multiple introductions from the Martinique, Guadeloupe, St. Martin and French Guiana, with a risk of autochthonous transmission if introductions occur during the warm season (Lazarus et al., 2016). *Ae. albopictus* was first detected in France in 2004; it is now well established in the south of the country (Romiti et al., 2026), where it has been responsible for local transmission of dengue and Chikungunya virus infections (Simonin, 2025; Tegar et al., 2026). *Ae. aegypti* was not yet reported in Continental France. Other imported infections occurred in Norway, Netherland, Denmark, Finland, Austria, Switzerland, Israel, Spain, Ireland, Sweden, England and Portugal (Jupille et al., 2016). While there were no secondary transmission cases, as none of the mosquito species that are possible vectors of Zika virus and other flaviviruses circulate in most of these countries, a certain risk of local transmission is in Spain, where *Ae. albopictus* is endemic.

Finally, in some regions, the viruses may adapt to new vectors. Thus, it has been suggested that *Ae. notoscriptus*, which is a competent vector of Chikungunya and Ross River viruses in Australia (Calvet et al., 2016), could function as a potential vector of Zika virus in New Zealand. This mosquito is present in Wellington province and is an experimentally competent vector of dengue and Japanese encephalitis viruses.

Climate change also affects vector efficiency in transmitting diseases. The extrinsic incubation period for dengue virus and other related alpha and flaviviruses (the time interval between pathogen ingestion by the vector and the moment when the vector becomes infective) is inversely correlated with the ambient temperature (Adepoju et al., 2023). Considering that an infected vector only becomes competent (i.e., capable of transmitting the pathogen) if the multiplication rate of the pathogen is fast enough and the vector survival time is long enough to permit the vector to become effective, a higher temperature can accelerate pathogen production (de Souza and Weaver, 2024). The egg-to-adult survival of *Ae. aegypti* (which is a vector for multiple viral infections) is 0% at 15 °C, increases up to 90% at 20 °C, followed by a slow decrease to 60% at 35 °C. Similarly, maturation from egg to adult takes 60 days at 15 °C, decreasing to 12 days at 20 °C and to ≈6 days at 27–34 °C. Finally, while the ability of mosquitoes to complete a blood meal in half an hour after a host becomes available is ≈50% of at 22-28 °C, it declines to virtually 0% at 33 °C (de Souza and Weaver, 2024). Altogether, these data provide a plausible explanation for the observed patterns of pathogen transmission. For example, cooler ambient temperatures limit the geographic range of *Ae. aegypti* and other vectors, pathogen transmission by the *Ae. aegypti* being more effective in the subtropical areas (i.e., South of Italy), than in the very hot areas surrounding the Equator (Kraemer et al., 2019; de Souza and Weaver, 2024). Meanwhile, the dynamics of the diseases produced by pathogens transmitted by vectors with aquatic developmental stages can also be driven by the precipitation patterns, and can dramatically change with climate alterations (Weaver, 2013; Paz, 2019; de Souza and Weaver, 2024). Heavy rainfall and prolonged drought can also dramatically impact the spatial-temporal distribution of vectors (Paz, 2019; D'Amore et al., 2022; Adepoju et al., 2023; de Souza and Weaver, 2024).

West Nile Virus (WNV) is a vector-transmitted virus that re-emerged over the last 30 years in both Europe and North America (Nash et al., 2001; D'Amore et al., 2022). The WNV vector is the *Culex* spp. mosquito, which is the common mosquito species in temperate regions, being dominant throughout North America, Europe and Asia. The recent WNV re-emergence was not due to vector habitat expansion, but to changes in WNV transmission triggered by changes in the temperatures in the mosquito’s endemic areas that led to increased rates of reproduction and incubation of the pathogens within the vector, and increased bite rates (Wang HR. et al., 2024). These were associated with an expanded duration of exposure to the vectors and implicitly to the pathogens due to the longer warm seasons (Wang H. et al., 2024).

Increased precipitation regimens may allow survival of *Ae. aegypti* at cooler temperatures, permitting it to expand its ecosystem to higher altitudes in Southern Europe, at the North of the Indian Subcontinent, or the highlands of Kenya and the Andes (Outammassine et al., 2022). This aspect is particularly important: historically, the higher altitude of all these regions compared to the Southern United States made it easier to implement public health measures to protect against the spread of vector-borne transmitted tropical diseases, in spite of the fact that Southern Europe is closer to the tropics than Southern United States (D'Amore et al., 2022; de Souza and Weaver, 2024). However, with global warming, mosquitos and viruses can thrive easier at higher altitudes, during a longer transmission season. This explains the numerous outbreaks of *Ae. albopictus* – transmitted chikungunya in Southern Europe (Italy and France) over the last two decades (Rocklov et al., 2019), and the postulated increase suitability for malaria and dengue to occur in the near future in the subtropical higher altitude areas of the Eastern Mediterranean region of Southern Europe, the Himalayas, the highlands of African nations (Kenya, Zambia) or in the Andes (Colón-González et al., 2021; Outammassine et al., 2022; Adepoju et al., 2023).

## Modeling the impact of climate change on epidemic diseases

There are numerous attempts to quantitatively assess the impact of climate change on the incidence and prevalence of infectious diseases, particularly its driving effect on epidemic outbreaks. In the past, some of the most consequential epidemics were driven by climate: thus, Justinian Plague is reported to have been exacerbated by repeated massive volcanic eruptions that occurred in 536, 540, and 547 AD and generated an unstable, cold climate, labelled as the “*Late Antique Little Ice Age*” (Preiser-Kapeller et al., 2025). Meanwhile, the cocolitzl epidemics, produced by the *Salmonella enterica* brought to Central America by the conquistadores were fueled by episodes of severe draught (Acuna-Soto et al., 2002; Acuna-Soto et al., 2004; Vagene et al., 2018). Conversely, heavy rainy periods and flooding fueled cholera outbreaks (Rieckmann et al., 2018). Therefore, Geographic Information System (GIS), a computer-based framework that can acquire, manage, analyze, and visualize spatial data tied to specific real-world locations is widely used for integrating climate-related diseases and outbreak mapping, with the goal of creating interactive maps, identifying hidden patterns, relationships, and spatial correlations between climatic variables (e.g., rainfall, temperature, humidity, flooding extent) and reported disease outbreaks (Cuadros et al., 2024; Javed et al., 2024; Pastula et al., 2025). Such correlations are currently used to identify potential linkages between environmental stressors and public health outcomes. These analyses objectivated several clear association between climatic variables and diseases. Thus, floodings in the high-risk zones can increase dengue incidence by 0.37% with each day of flooding, as identified through spatial clustering, which can permit proactive, targeted vector control (Hornyak, 2017).

Use of GIS tools to guide malaria control efforts in response to severe flooding found a disproportionate increase in the risk of malaria in the areas that appeared most affected by flooding (Xu et al., 2024). Use of GIS is necessary to improve measure of malaria control, as the case incidence rates have flattened in the recent years, after a constant reduction that followed the unprecedented investment in malaria control started in the early 2000s. These trends are due to the rapid growth of the populations at risk and to the increasing climatic shocks (Weiss et al., 2025). Use of the geotemporal model linked to an ensemble of climate projections under the Shared Socioeconomic Pathway 2-4.5 (SSP 2-4.5) scenario (Symons et al., 2026), led to estimates that, by 2050, climate change could contribute to 123 million additional malaria cases and 532,000 additional deaths in Africa if the control approaches are unchanged (Symons et al., 2026). Extreme weather is the prime driver of this increased risk, being responsible for 79% of additional cases and 93% of additional deaths, calling for climate-resilient malaria control strategies and robust implementation (Symons et al., 2026). The geotemporal model also pointed to an intensification of malarial activity in the current endemic areas rather than range expansion (Symons et al., 2026).

With regard to the measures for forecasting cholera dynamics, a CholeraMap was developed using high‐resolution earth observations. CholeraMap disseminates geocoded risk maps directly to Matlab’s population via a mobile smartphone application. This approach avoids relying on station‐based environmental and hydroclimatological data, which are difficult to obtain, instead uses remote sensing data sets for designing and operating early warning systems for epidemic diseases (Nusrat et al., 2024).

Use of GSI and CholeraMap permitted several important observations. Thus, in Zanzibar, during the monsoon season, a rise of 200 mm of rainfall associated a 1.6‐fold increase in cholera cases, following a 2‐month lag (Reyburn et al., 2011). Conversely, in Yemen, which has an arid subtropic climate, a positive association was reported between weekly rainfall and a shorter lag period for cholera of about 10 days (Camacho et al., 2018). This is due to the fact that a lower rainfall drives an increase of pathogen and wastewater elements concentration in water sources, reducing the quality of water and increasing the risk of human contact with pathogens (Moors et al., 2013; Levy et al., 2016).

Temperature also impacts the infectious diseases outbreaks. Pathogens develop and reproduce optimally at 31 ± 3 °C, this being the temperature associated with the highest incidence of cholera leading to higher cholera occurrence (Silva and Benitez, 2016; Asadgol et al., 2019; Saha et al., 2019). Furthermore, temperature increases result in accelerated evaporation, and lower water bodies. Increases of pathogen concentration due to water shortage lead to cholera outbreaks (Jutla et al., 2013).

As such, approaches to overly climate data (temperature, humidity, rainfall, flooding) with social vulnerability indexes allow us to better circumscribe high-risk areas. Use of tools (i.e., CDC Heat & Health Tracker) (C.D.C. Centers for Diseases Control and Prevention, 2023) to analyze these interactions to improve public health responses. Such tools provided useful data demonstrating that that rising temperatures increase cardiovascular, respiratory, and direct heat-related illnesses.

## Charting the future: uncertainties related to climate change research

While the scientific consensus on anthropogenic climate change is robust (Knight and Harrison, 2013; Tian et al., 2016; Lynas et al., 2021; Gong et al., 2024), a degree of uncertainty remains regarding the precise magnitude and regional expression of future climate impacts. These uncertainties arise from several sources: limited understanding of the complex interactions within Earth’s systems (e.g., cloud formation, ocean circulation, and aerosol feedbacks) (Douville et al., 2022; Schwarzwald and Lenssen, 2022), measurement variability, and the unpredictable influence of human behavior on emissions trajectories (Paz, 2019). More recently, advances in climate modeling employed dimensionality-reduction and clustering techniques to improve the reliability of regional-scale projections, addressing part of the uncertainty challenge (Achour et al., 2024).

Despite limitations, climate models have consistently reproduced key trends observed over the past century, including the rate of atmospheric and oceanic warming, the frequency of extreme weather events, and the acceleration of ice melt (Cornwall, 2019a; Cornwall, 2019b). Their predictive reliability is supported by multiple independent datasets, such as ice cores, tree rings, and satellite measurements (WHO; I.P.C.C. The Intergovernmental Panel on Climate Change, 2014; Vaidyanathan, 2014; Hoegh-Guldberg et al., 2018; Supran et al., 2023; Jackson and Encyclopedia Britanica, 2024; Mann and Encyclopedia Britannica, 2024).

Importantly, uncertainty in climate projections should not be interpreted as a reason for inaction. As the range of possible outcomes includes both moderate and severe scenarios, the precautionary principle suggests that proactive mitigation and adaptation strategies remain essential to safeguard global health and environmental stability (Knight and Harrison, 2013; Douville et al., 2022; Schwarzwald and Lenssen, 2022).

## Climatic skepticism

Climate change skeptics argue that the reported global warming is nonexistent and that, with climate dynamics being cyclic, the alterations that we observe now are in fact just a peak in the rising temperature cycle, being likely that a cooling of the planet will follow (Vaidyanathan, 2014). These denialists argue that increases in the CO_2_ levels are not a cause, but a consequence of global warming, and that carbon cycles are too vast to upset nature’s balance (Cook, 2016). Natural cycles of warming and cooling, such as the Holocene Climate Optimum (~9,500–5,500 years ago), illustrate that climate variation is part of Earth’s long-term dynamics. However, the current anthropogenic warming is far more rapid and intense than any natural trend observed in the past 10,000 years.

Climate change denial arguments were made continuously over the last 50 years by both scientists and political activists which can be traced to the fossil fuel industry, political, and ideological interests (Vaidyanathan, 2014). Their overall goal is to undermine the public trust by creating a false appearance of a scientific controversy. In reality, there is an overall consensus between scientists (>98%) that human activities are responsible for global warming (Lynas et al., 2021). For comparison, only 80% of scientific studies agree that there is a relation between the sodium intake and high blood pressure (Neal et al., 2013) and only 87% of scientists consider that evolution is due to natural selection (Pew Research Center, 2009).

The main counterargument to climate denialists is that, irrespective of the nature of the warming period, such periods are not really transient. The Holocene Climate Optimum lasted approximately 4,000 years, i.e., tens of generations. As such, regardless if whether the temperature increase triggered by human activities or due to a cyclic evolution, the adverse health impacts of the warming periods will require comprehensive mitigation and adaptation strategies. Climate adaptation is unavoidable to counter the health risks inherent to these changes. Archaeological evidence suggests that climatic fluctuations in prehistory may have contributed to localized disease outbreaks, such as the epidemic that decimated the Neolithic settlement of Hamin Mangha in northern China (Ji et al., 2014). Such associations highlight how shifts in environmental stability can alter human disease dynamics. Similarly, throughout history, abrupt climate alterations (whether cooling or warming) have often coincided with major epidemic events (Acuna-Soto et al., 2002; Acuna-Soto et al., 2004; Rieckmann et al., 2018; Vagene et al., 2018; Preiser-Kapeller et al., 2025).

Historical records show that extreme climatic events, such as prolonged droughts or floods (Rieckmann et al., 2018; Vagene et al., 2018; Luterbacher et al., 2020), have repeatedly preceded major epidemics, including outbreaks of plague in medieval Europe (Luterbacher et al., 2020), epidemics in 16th-century Mexico (Acuna-Soto et al., 2002; Acuna-Soto et al., 2004; Vagene et al., 2018), and waterborne diseases like cholera (Rieckmann et al., 2018). These historical parallels underscore that climate disruptions have long influenced pathogen dynamics and human health (Hays, 2005).

## Policy recommendations to minimize the impact of climate change on human health

To limit the impact of the climate change on human health, robust and sustained plans to control the global warming are necessary. The Paris agreement, although not very ambitious, was supposed to limit the CO_2_ emissions and stop the accelerated trends of the last 50 years. Yet, not all the countries signed that agreement, and the USA withdraw from the Paris agreement for few years and again in 2025.

Multiple other government expert panels identified the magnitude of the problems and proposed actions to counter climate change. Thus, the Intergovernmental Panel on Climate Change Fifth Assessment Report (IPCCAR5) identified the three major ways through which climate change negatively impacts human health: (a) extreme weather conditions, (b) disease vectors, water-borne diseases, and air pollution, and (c) human-made socio-economic drivers: occupational factors, undernutrition, and mental stress (I.P.C.C. The Intergovernmental Panel on Climate Change, 2014). The report stresses that lack or cut-back in public infrastructure, such as health care, may result in the emergence/re-emergence and spread of infectious diseases triggered by the global warming (I.P.C.C. The Intergovernmental Panel on Climate Change, 2014). The report also points at Southern Europe and the south of the United States as the areas that will be most likely affected by health issues related to climate change.

Another reflection group, the Intergovernmental Panel on Climate Change Special Report (IPCCSR) stated that the global temperature levels 1.5 °C higher than the preindustrial levels increase vulnerability to diseases and reduce the capacity of human health systems to manage such climate related impacts (Hoegh-Guldberg et al., 2018). The report attributes temperature increases to global greenhouse gas emissions (Hoegh-Guldberg et al., 2018). To counter the negative impacts of global warming, the panel recommended strengthening the global response and promotion of sustainable development activities. Given the high probability of increase in the global spread of vector-borne diseases (especially in climate hot spots), the panel recommended emergency public health measures as a form of adaptive action to prevent or reduce morbidity and mortality from vector-borne diseases. Recent complementary analyses such as an agroclimatic zoning of Brazil’s Minas Gerais region, where rising water deficits after 2060 are projected to limit sugarcane viability despite mechanization potential illustrate how climate adaptation must integrate agriculture and land-use planning into health policy frameworks (de Carvalho Júnior et al., 2025). While the IPCCSR report is not very specific, public health adaptation measures could involve, but not being limited to: (a) strengthen the healthcare system to foresee, diagnose and react to climate-related crises and risk; (b) adapt the policies to the emerging risks, while creating an infrastructure able to rapidly react and accommodate such crises. For example, multiple public health systems implemented wastewater testing for pathogens as an effective tool for communities to act fast to prevent disease spread in communities (Mashau et al., 2024). This sentinel could be used, and it is actually widely implemented to monitor the emergence and re-emergence of human pathogens (Girón-Guzmán et al., 2024; Grassly et al., 2025; Singh et al., 2024); (c) prepare the healthcare workforce to deal with new diseases that need to be properly diagnosed and treated; (d) implementation of early-warning systems, and of well-defined plans to deal with climate calamities. Such plans should, for example include actions for providing care in areas damaged by hurricanes and flooding, when the entire system is subjected to multiple stressors. An increased focus on climate change training is currently in operation at the University of Pittsburgh’s School of Public Health (https://www.publichealth.pitt.edu/research-practice/priority-areas).

Particular efforts should be directed to educate communities at risk for health care crises triggered by the effects of the global warming. Particularly, persons located in areas at risk for acute climate crises (i.e., areas prone to flooding, or that could be impacted by the global sea level raise; hurricane/typhoon-prone areas; areas at risk for extensive wildfires) should have very clear contingency plans to deal with such crises. Evacuation plans would probably be desirable; should evacuation not be doable, very clear plans for survival and for health conservation need to be in place.

Since virtually every person on the planet will be impacted by the temperature raise, communities should be systematically instructed on how to cope with climate change (better hydration, better sunscreen protection, plans for saving the crops and maintaining the food chains etc.).

Finally, public preparedness should start from the axiomatic truth that climate change is not temporary and that they can actually last for millennia, and that we have to prepare iterative approaches to address these health risks over long term (Haines and Ebi, 2019). Such adaptation will likely involve irreversible changes in human behavior and adaptation to the temperature increase and its consequences (extreme heat or sea level rises). Some of these adaptations which will help managing climate-related health risks need to come from outside the health care system (i.e, ensuring water and food security) (Myers et al., 2014). One aspect to be considered is that climate adaptations may result in political crises, as studies suggested that temperature fluctuations are related to the increases in asylum applications both in Europe and in the United States (Missirian and Schlenker, 2017). These aspects should be addressed in close collaboration with the policy makers (i.e., relevant ministries and departments and the public health experts). Every strategic plan should be proofed to promote health while preventing unintended adverse consequences (i.e., poverty increase due to excessive spending aimed at protection).

There is a general consensus that countering the deleterious impact of climate change on public health requires actions going beyond general policies and implemented at every level of the society. Scientists released multiple calls for action. In December 2023, around the time of the 28^th^ UN Conference of the Parties (COP) on climate change, which was held in Dubai, more than 200 medical journals published simultaneously a call to the UN, political leaders, and health professionals to consider an unified view that climate change and biodiversity loss is a single indivisible crisis, calling to sustain common actions targeting these crises, in an attempt to control and reverse them (Abbasia et al., 2023). These articles were written in support of the conclusion of a common workshop held in 2020: “only by considering climate and biodiversity as parts of the same complex problem … can solutions be developed that avoid maladaptation and maximize the beneficial outcomes” (Otto-Portner et al., 2021).

It was proposed that, in order to properly address the issues of climate change, research has to be conducted in agreement with the principles of responsible research and innovation (RRI) (Ligardo-Herrera et al., 2018). RRI offers a framework for aligning scientific progress with societal needs. Applying RRI principles such as transparency, ethical conduct, and public engagement can enhance the credibility and impact of climate-health research and policy.

A major feature of the climate change is that it will disproportionately impact the children and young adults of today. These generations will likely have to face serious issues related to global warming throughout their lives and careers. They will likely, and hopefully, be the ones to implement all the measures listed in the current plans for action. However, as revealed over the last few decades, climate change due to global warming and their overall consequences might sometimes be unpredictable, or they may evolve at a different pace than initially modeled. This is a call for a thorough and firm action from generational actors. Climate change and global warming should be central aspects of current education, and educational programs should be implemented at every societal level. We need to acknowledge that the current targets may be insufficient to address the main problems, and thus new, more ambitious, goals might need to be considered in the near future. For example, while electric vehicles have a substantially lower carbon foot print, they are still employing a lot of energy. Reducing car use is thus a priority that can be achieved with improved public transportation, use of individual transportation vehicles without a carbon foot print (i.e., bicycles and scooters), and other measures that improve access with limited impact on climate. Solving the complex challenge of climate variability will necessitate a coordinated and sustained global action, independent of political views, geographical location and individual interests.

## Conclusions

Human-driven climate change has become one of the most important threats for global health worldwide, creating stress conditions that have the potential to generate new pathology and increase the prevalence and severity of several common diseases. One of the most dramatic medical features of climate change is the production of ecological imbalances that expand the habitat and persistence of vectors of pathogens resulting in the emergence of diseases in previously nonendemic regions worldwide. As all the scientific and government panel’s report that the process of global warming will continue, national, regional and global level policies are needed to address the potential health and economic hazards of climate change. Worldwide implementation of the principles of responsible research and innovation has a real potential to improve climate conditions and halt current changes, if adopted and conducted worldwide.
